# Enhanced Nitric Oxide (NO) and Decreased ADMA Synthesis in Pediatric ADHD and Selective Potentiation of NO Synthesis by Methylphenidate

**DOI:** 10.3390/jcm9010175

**Published:** 2020-01-08

**Authors:** Kathrin Jansen, Beatrice Hanusch, Saskia Pross, Erik Hanff, Kathrin Drabert, Alexander Bollenbach, Irina Dugave, Christina Carmann, Rainer Georg Siefen, Barbara Emons, Georg Juckel, Tanja Legenbauer, Dimitrios Tsikas, Thomas Lücke

**Affiliations:** 1 University Children’s Hospital, Ruhr University Bochum, 44791 Bochum, Germany; 2 Children’s Hospital, St., Clemens-Hospital Geldern, 47608 Geldern, Germany; 3 Institute of Toxicology, Core Unit Proteomics, Hannover Medical School, 30625 Hannover, Germany; 4 Department of Psychiatry, Alexius/Josef Hospital, 41464 Neuss, Germany; 5 Department of Psychiatry, LWL Institute of Mental Health, LWL University Hospital, Ruhr-University Bochum, 44791 Bochum, Germany; 6 Department of Psychiatry, LWL University Hospital, Ruhr-University Bochum, 44791 Bochum, Germany; 7 LWL University Hospital Hamm for Child and Adolescent Psychiatry, Psychotherapy and Psychosomatic, Ruhr University Bochum, 59071 Hamm, Germany

**Keywords:** L-Arg/NO pathway, cardiovascular risk, psychiatric disorder, pharmacotherapy, blood pressure regulation

## Abstract

Attention deficit hyperactivity disorder (ADHD) is a common pediatric psychiatric disorder, frequently treated with methylphenidate (MPH). Recently, MPH’s cardiovascular safety has been questioned by observational studies describing an increased cardiovascular risk in adults and blood pressure alterations in children. We considered members of the L-arginine (Arg)/nitric oxide (NO) pathway as possible early cardiovascular risk factors in pediatric ADHD children. They include the NO metabolites, nitrite and nitrate, the NO precursor Arg, and asymmetric dimethylarginine (ADMA), an endogenous NO synthase (NOS) inhibitor and a cardiovascular risk factor in adults. We conducted a prospective clinical trial with 42 ADHD children (aged 6–16 years) with (*n* = 19) and without (*n* = 23) MPH treatment. Age-matched children without ADHD (*n* = 43) served as controls. All plasma and urine metabolites were determined by gas chromatography-mass spectrometry. We observed higher plasma nitrite and lower plasma ADMA concentrations in the ADHD children. MPH-treated ADHD children had higher plasma nitrite concentrations than MPH-untreated ADHD children. As NOS activity is basally inhibited by ADMA, MPH treatment seems to have decreased the inhibitory potency of ADMA. Percentiles of systolic blood pressure were higher in MPH-treated ADHD children. The underlying mechanisms and their implications in the MPH therapy of pediatric ADHD with MPH remain to be elucidated in larger cohorts.

## 1. Introduction

Attention deficit hyperactivity disorder (ADHD) is a common psychiatric disorder in childhood, globally affecting about 2%–7% of all children [1]. Although ADHD is recognized as a typical pediatric disease, symptoms can maintain until adulthood [2,3]. ADHD is characterized by impulsivity and/or inattention and hyperactivity associated with alterations in the catecholaminergic system, such as dopamine or norepinephrine homeostasis [4]. Stimulant medication, such as the use of methylphenidate (MPH), is recommended as pharmacotherapy. MPH’s mode of action is based on dopamine transporter inhibition, which leads to increased synaptic dopamine concentrations thereby mitigating ADHD symptoms [5]. Although MPH is considered an effective first-line drug, there is some uncertainty about the cardiovascular safety of this drug. A number of population-based observational studies reported an increased cardiovascular risk, e.g., hypertension and heart rate elevation in adults [6,7,8,9]. However, MPH was found to be a vasodilator in dogs [10]. The cardiovascular risk of stimulant medications seems not to be as pronounced for children as for adults. Yet, recent meta-analyses revealed elevated blood pressure and heart rate both in children and in adolescents treated with MPH, albeit smaller than in adults [11,12]. Consequently, long-term consequences of MPH-medication in childhood have to be considered. It is, therefore, important to identify specific biomarkers to monitor potential cardiovascular side-effects caused by MPH-treatment. 

**Figure 1:**
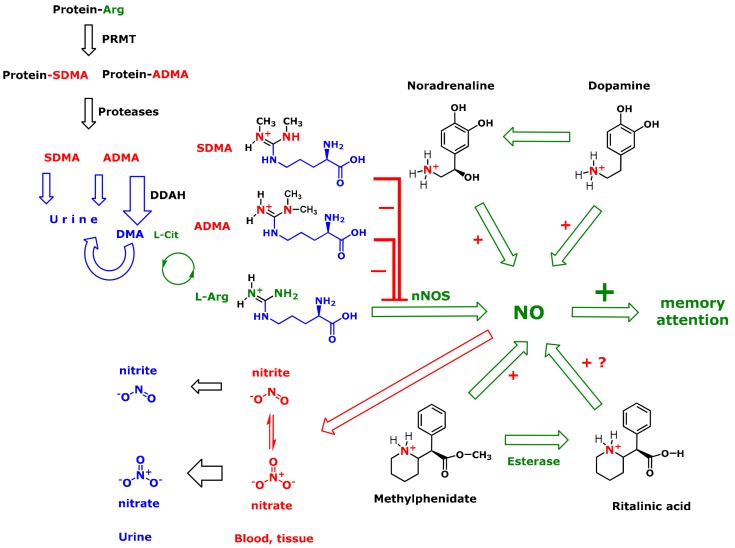
Simplified schematic of the L-arginine/nitric oxide pathway in the attention deficit hyperactivity disorder, its interaction with methylphenidate, and possible effects on memory and attention. The symbol − means inhibition, reduction, or attenuation. The symbol + means activation or enhancement. For more details see the text. ADMA, Asymmetric dimethylarginine; Arg, Arginine; DDAH, Dimethylarginine dimethylaminohydrolase; DMA, Dimethylamine; L-Cit, L-Citrulline; NO, Nitric Oxide; nNOS, Neuronal nitric oxide synthase; PRMT, Protein arginine methyltransferase; SDMA, Symmetric dimethylarginine

The L-arginine (Arg)/nitric oxide (NO) pathway is virtually present in all cell types, including endothelial, neuronal, and immune cells. Arg is the substrate of all known NO synthase (NOS) isoforms. This enzyme family converts Arg to NO and L-citrulline (Figure 1). The physiological functions of NO are manifold and include vasodilatation, inhibition of platelets and leukocyte adhesion, and neurotransmission [13,14]. A dysregulated Arg/NO pathway is associated with endothelial dysfunction and hypertension [15]. Thus, we hypothesized that the Arg/NO pathway is altered in newly diagnosed and MPH-treated ADHD children (Figure 1). The status of the Arg/NO pathway in health and disease can be determined by measuring the concentration of certain members of this pathway in blood and urine. NO itself is a very short-lived molecule and analytically inaccessible. Under certain conditions, circulating and urinary major metabolites of NO, i.e., nitrite and nitrate, are suitable surrogates of NO synthesis [16]. Thereby, it is worthy to mention that there is a nitrate/nitrite/NO cycle by which NO can be generated independently of NOS [17] (Figure 1). Hemoglobin and myoglobin, as well as xanthine oxidoreductase and mitochondrial cytochrome c oxidase, can convert nitrate and nitrite to NO during hypoxia. Furthermore, cytochrome P450 and cytochrome P450 reductase are able to release NO from nitrate and nitrite in the endothelium [18].

The endogenous *N*-guanidine (*N*^G^) methylated Arg analogs *N*^G^-methyl-L-arginine (L-NMMA), asymmetric dimethylarginine (ADMA), and symmetric dimethylarginine (SDMA) are inhibitors of NOS [19,20]. These *N*^G^-methylated arginines are produced by regular hydrolysis of certain proteins methylated on Arg residues by protein arginine methyltransferases (PRMT) [21] (Figure 1). About 80% of daily produced ADMA are intracellularly hydrolyzed to dimethylamine (DMA) and L-citrulline by dimethylarginine dimethylaminohydrolase (DDAH) [20]; DMA is excreted in the urine, whereas L-citrulline is further metabolized to Arg (Figure 1). Expression and activity of DDAH are reduced by oxidative stress caused by shear stress, tumor necrosis factor α, oxidized low-density lipoprotein, and homocysteine [22]. On the contrary, antioxidant mediators, such as estradiol and interleukin-1β (IL-1β) increase DDAH expression [20,22]. In adults, ADMA has emerged as a cardiovascular risk factor [23]. High ADMA plasma concentrations were found to correlate with endothelial dysfunction, hypertension, and atherosclerosis, among others [24,25,26]. In children with hypertension, higher circulating ADMA concentrations were found compared with normotensive children [27]. Yet, it is uncertain whether ADMA is a cardiovascular risk factor in children. It is worth mentioning that the Arg/NO pathway in children and adolescents differs from that in adults. Especially, ADMA synthesis is much higher in healthy children and adolescents compared to healthy adults [28].

The aim of the present study was to determine the status of the Arg/NO pathway in children with ADHD, especially focusing on ADMA and nitrite. We included in the study ADHD children without MPH treatment (i.e., newly diagnosed with ADHD) and ADHD children treated with MPH to observe potential effects of MPH on the Arg/NO pathway. Healthy children without ADHD served as controls.

## 2. Methods

### 2.1. Subjects

The study was designed as a prospective clinical trial. Forty-two subjects diagnosed with ADHD were included. All patients met the Diagnostic and Statistical Manual of Mental Disorders IV (DSM-IV) criteria. Of these, 23 children were not treated with MPH (−MHP group), and 19 children were treated with MPH (+MPH group). The time window from ADHD diagnosis to the start of the study was 6.6 ± 14.0 months for the −MPH group and 9.4 ± 14.6 months for the +MPH group. For two subjects of the +MPH group, the information for the initial diagnosis was missing. Dosages for MPH varied from 10 to 50 mg per day (20.8 ± 13.1 mg/d). Forty-three healthy children (15 females, 28 males; aged 9.7 ± 2.6 years) receiving elective surgery served as controls. Exclusion criteria were epilepsy, mental disability, intelligence quotient (IQ) < 50, and chronic or endocrinological diseases. Systolic blood pressure (SBP) and diastolic blood pressure (DBP) were measured using the Riva–Rocci method. Age-, sex-, and height-specific BP percentiles were calculated for SBP and DBP. Mean arterial pressure (MAP) was calculated using the formula: (2 × DBP + SBP)/3. The anthropometric characteristics of the ADHD children are presented in Table 1. The study was approved by the Ethics Committee of the Ruhr-University Bochum. Written informed consent was obtained from the parents of all individual participants included in the study. 

### 2.2. Sampling and Biochemical Analyses

Venous blood samples were collected in ethylenediaminetetraacetic acid (EDTA) monovettes (5 mL). Plasma was generated by centrifugation (4000× *g*, 10 min, 4 °C). Midstream urine samples were collected in the morning. Plasma and urine samples were stored at −80 °C until further analysis. Nitrate, nitrite, and creatinine in plasma and urine samples were determined simultaneously by gas chromatography-mass spectrometry (GC-MS) as described before [28]. Arg and ADMA in plasma and urine were analyzed with GC-MS as described elsewhere [29]. Urinary SDMA and DMA were measured by GC-MS as described [30,31]. The urinary excretion of nitrite, nitrate, Arg, ADMA, SDMA, and DMA was corrected for creatinine excretion and these data are reported as µM analyte per mM creatinine (Crea). The nitrate-to-nitrite molar ratios in plasma (P_NOx_R) and urine (U_NOx_R) were determined by dividing the concentration of nitrate in plasma (P_NO3_) or urine (U_NO3_) by the concentration of nitrite in plasma (P_NO2_) or urine (U_NO2_) (P_NOx_R = P_NO3_/P_NO2_; U_NOx_R = U_NO3_/U_NO2_). U_NOx_R, P_NOx_R, and their ratio U_NOx_R/P_NOx_R are considered nitrite-dependent renal carbonic anhydrase activity [32]. The fractional excretion (FE, %) of nitrite and nitrate was calculated using the following formula: FE (%) = (U_NOx_ × P_Crea_)/(P_NOx_ × U_Crea_) × 100.

### 2.3. Statistical Analyses

Statistical analyses were performed using the statistical software package IBM^®^ SPSS^®^ Statistics for Windows, version 25.0 (IBM Corp., Armonk, NY, USA) and GraphPad Prism 7. Descriptive data were analyzed by the Chi-squared test. Normally distributed data were analyzed using parametric tests (Student’s *t*-test, one-way ANOVA). Non-normally distributed data or data without variance homogeneity were analyzed using non-parametric tests (Mann–Whitney test, Kruskal–Wallis test) and Bonferroni post hoc analysis. Values of *p* < 0.05 were considered significant. Data are presented as mean ± standard deviation (SD) or median [25th–75th interquartile range].

## 3. Results

Regarding age or gender distribution, no differences were seen between the +MPH and −MPH groups (Table 1). Age (9.6 ± 2.6 years) and gender (15 females, 34.9%) distribution of controls were comparable to those of the ADHD children as well (*p* = 0.34 and *p* = 0.39, respectively). The anthropometric characteristics of the ADHD children of the +MPH and −MPH groups showed a minor but statistically significant difference with respect to the head circumference, which was smaller by 1.7% in the +MPH group. SBP percentiles were by 29% higher in the +MPH group compared to the −MPH group (*t* (39) = 1.58; *p* = 0.03, Table 1), while MAP was not different between the groups. 

**Figure 2:**
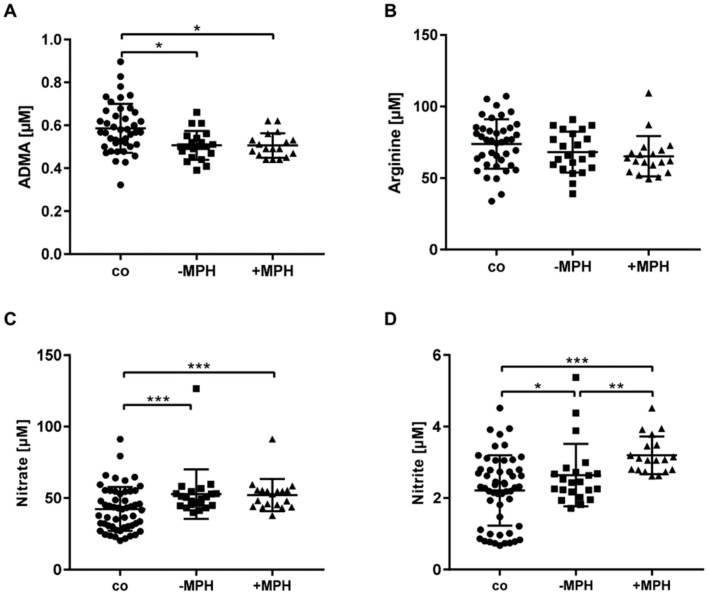
Plasma concentrations of asymmetric dimethylarginine (ADMA) (**A**), arginine (**B**), nitrate (**C**), and nitrite (**D**) of non-ADHD control children (co) and in ADHD children without (−MPH) and with methylphenidate (+MPH) treatment. * *p* < 0.05, ** *p* < 0.01, *** *p* < 0.001.

The plasma concentrations of the analytes are displayed in Figure 1 (see also Table S1). The plasma concentration of Arg did not differ between age- and gender-matched children without ADHD (control group) and the two ADHD groups (Figure 2). The ADMA plasma concentrations in controls were higher compared to ADHD children (*p* = 0.001). There was no difference between −MPH and +MPH for age-corrected ADMA plasma concentrations of the ADHD children (see Table S1). The plasma nitrite concentration was higher in the +MPH group compared to the −MPH group (*p* = 0.006). This difference persisted after correction of the plasma nitrite concentration by age of the ADHD children (see Table S2). The control group showed lower plasma nitrate and nitrite concentrations compared to the −MPH (*p* < 0.001; *p* = 0.01) and +MPH (*p* < 0.001; *p* < 0.001) groups.

The age-corrected ADMA and nitrite plasma concentrations of the ADHD children were divided into two groups according to their IQ values ranging between 50 and 100 (−MPH 50–100, +MPH 50–100) and IQ values above 100 (−MPH > 100, +MPH > 100). We found no interaction of MPH and IQ with regard to age-corrected nitrite plasma concentrations, but we found an interaction for age-corrected ADMA plasma concentrations (Table S3). Without medication, ADMA plasma concentrations were higher in children with IQ > 100, while ADHD children treated with MPH showed lower ADMA plasma concentrations in children with IQ > 100 when compared to +MPH 50–100 (Table S3).

Creatinine-corrected urinary nitrate and nitrite concentrations in the control group did not differ from those in the −MPH and +MPH groups (Table 2). Also, the creatinine-corrected excretion rates of ADMA, SDMA, DMA, ADMA+DMA+SDMA, (ADMA+DMA)/SDMA, and Arg were not different in the −MPH and +MPH groups (Table 2). The sum of ADMA, DMA, and SDMA (i.e., ADMA+DMA+SDMA) represents the whole-body Arg-dimethylation in proteins, and the molar ratio (ADMA+DMA)/SDMA is a measure for the balance between asymmetric and symmetric whole-body Arg-dimethylation in proteins [31].

The molar ratio of plasma nitrate (P_NO3_) to plasma nitrite (P_NO2_) concentration, i.e., P_NOx_R and the molar ratio of urinary nitrate to urinary nitrite (i.e., U_NOx_R) did not differ between the ADHD groups (Table 3). There was no statistical difference between the ADHD groups with respect to U_NO3_/P_NO3_, but U_NO2_/P_NO2_ was lower in the ADHD children compared to the non-ADHD children. The U_NOx_R/P_NOx_R tended to be increased in ADHD children, especially in the +MPH group compared to the control group. The fractional excretion (FE) of nitrate (FE_NO3_) was about 15% in both ADHD groups. The fractional excretion of nitrite (FE_NO2_) was 0.6% in the −MPH group and 0.4% in the +MPH group (Table 3).

## 4. Discussion

The aim of this study was to investigate the status of the Arg/NO pathway (Figure 1) in newly diagnosed, untreated ADHD children (−MPH group), and in ADHD children treated with MPH (+MPH), also addressing the question of a potential cardiovascular risk in ADHD in terms of hypertension and the influence of MPH. 

There is solid evidence that the Arg/NO pathway is involved in a variety of physiological and pathological conditions such as cardiovascular diseases, but also in Alzheimer’s disease and thyroid disorders in adults [33,34,35,36,37]. In recent years, the Arg/NO pathway is increasingly investigated in childhood and adolescence. Investigations show that there is a remarkable difference between adults and children with respect to the Arg/NO pathway [38]. Biomarkers such as ADMA, which are recognized as cardiovascular risk factors in adults, may not necessarily be cardiovascular risk factors in children and adolescents. As an example, circulating ADMA concentrations above 400 nm are considered to be associated with disease in adults, whereas healthy infants have much higher circulating ADMA concentrations, which decrease with age from about 1000 nm to reach about 400 nm at the age of about 18 to 20 years [38]. 

Studies on pediatric ADHD with respect to the Arg/NO pathway are very rare. In the present study, we investigated the Arg/NO pathway in two groups of ADHD children and in healthy non-ADHD children by measuring representative members of the Arg/NO pathway in plasma and urine samples. Nitrite and nitrate were chosen as indices of NO synthesis [16,39]. Arg and ADMA were chosen as the substrate and endogenous inhibitor of NOS activity, respectively [39]. The results of our study are discussed in detail as follows in the subsequent sections.

### 4.1. NO Synthesis is Elevated in Pediatric ADHD

ADHD children had higher circulating nitrate and nitrite concentrations, indicating higher systemic NO synthesis compared to children without ADHD. ADHD children had lower urinary nitrate and nitrite excretion rates compared to those of age-matched non-ADHD children. The most remarkable and statistically significant difference between the −MPH and +MPH ADHD groups was observed for plasma nitrite. Obviously, plasma nitrite levels are not only influenced by ADHD itself, but further increase after MPH treatment. On the contrary, an experimental approach showed that chronic MPH treatment of healthy rats did not cause any differences of nitrite plasma levels [40]. The fractional excretion (FE) values of nitrite and nitrate did not differ between the ADHD groups. This finding may suggest that ADHD is associated with higher systemic NO synthesis and that MPH is likely to have exerted a stimulating effect on systemic NO synthesis in the ADHD children treated with MPH. This seems not to be due to a higher bioavailability of Arg, as the circulating Arg concentrations were closely comparable in both ADHD groups and in the non-ADHD children. Additionally, the U_NOx_R, the molar ratio of urinary nitrate to nitrite did not differ between the groups. U_NOx_R is supposed to be a measure for the renal carbonic anhydrase-dependent reabsorption of nitrite. Carbonic anhydrase may also be responsible for NO formation in the presence of thiols such as L-cysteine and, thus, may determine the NO reservoir and bioavailability in the renal and cardiovascular systems [32]. Our study suggests that ritalinic acid, the major MPH metabolite, may have increased the reabsorption and/or decreased the excretion of urinary nitrite and nitrate by about 30% and 10%, respectively, thus eventually increasing the plasma nitrite concentration.

Circulating nitrite is considered to reflect NO synthesis by the two constitutive NOS isoforms [41], i.e., neuronal NOS (nNOS) and endothelial NOS (eNOS). Circulating nitrate and nitrite may also represent a considerable reservoir of NO bioactivity independent of NOS [17]. As all NOS isoforms may contribute to circulating nitrite, the plasma nitrite concentration does not provide dependable information of an individual NOS isoform [16]. In a mouse model of ADHD, MPH was shown to increase nitrite concentration and expression of eNOS mRNA and inducible NOS (iNOS) mRNA, but not of nNOS mRNA in the hippocampus [42]. Therefore, it cannot be excluded that MPH had induced iNOS expression/activity in the MPH-treated ADHD children. 

That the urinary concentrations of nitrite and nitrate were tightly comparable in the ADHD and the non-ADHD children, while their plasma concentrations differed significantly, suggests that an extra-renal synthesis of NO may be induced in ADHD patients and further be elevated by MPH. As nitrate may be converted in various compartments of the body to nitrite and, in turn, nitrite is a potent nitrosating species, elevation of circulating nitrate and nitrite concentrations by diet and disease may exert detrimental health effects. Given the physiological concentrations of circulating nitrate and nitrite both in ADHD and non-ADHD children and their relatively small MPH-induced increases, no detrimental effects by nitrate/nitrite are expectable in MPH-treated ADHD children.

### 4.2. ADMA Synthesis is Decreased and nNOS Inhibition by ADMA is Attenuated in Pediatric ADHD

In both ADHD groups, we measured almost the same ADMA concentrations in plasma and urine, respectively. The ADMA plasma concentration in the ADHD children is on average by 70 nm lower than in the non-ADHD children of the same age. Obviously, MPH treatment has no effect on ADMA synthesis, metabolism, and excretion. Higher ADMA concentrations may increase the extent of the intrinsic inhibition rate of NOS activity [19], while lower ADMA concentrations may attenuate the extent of inhibition of NOS activity [20]. Therefore, the higher systemic NO synthesis observed in the ADHD children compared to the non-ADHD children could be due to an attenuated inhibition of NOS activity as the result of the lower ADMA concentration prevailing in pediatric ADHD compared to non-ADHD children. ADMA is a much stronger inhibitor of nNOS activity (IC_50_ = 1.5 µM) than of eNOS activity (IC_50_ ≈ 12 µM) and has been suggested to be a modulator of nociception [43]. In addition, nNOS is a much more abundant NO-producer than eNOS [44]. Hence, it is reasonable to assume that the higher circulating nitrite concentrations measured in the ADHD children of our study are likely to arise from NO produced from Arg by the catalytic action of nNOS in neuronal cells (Figure 1). 

### 4.3. Potential Effects of MPH on NO and ADMA Synthesis in Pediatric ADHD

The results of our study may suggest that NO synthesis is elevated in neuronal cells due to locally decreased ADMA concentrations, eventually enhancing sensory hyper-responsiveness in a midbrain sensory structure (superior colliculus). This might be an underlying mechanism of distractibility in ADHD which increases inattention and distractibility in pediatric ADHD [45]. Amphetamine is a first-line agent in ADHD treatment that is connected to higher dopamine release and reduced reuptake in the brain [46]. In rat mesencephalic primary cultured neuron preparations, the experimental NO donor NOC-12 (i.e., 3-ethyl-3-(ethylaminoethyl)-1-hydroxy-2-oxo-1-triazene) increased dopamine release and potentiated the amphetamine-induced release of dopamine. Hence, amphetamine and NO donors might act in favor of dopamine release through different pathways [47]. The dopamine release induced by NOC-12 was found to depend on soluble guanylyl cyclase, which is activated by NO to produce cGMP and subsequently to stimulate protein kinase G activity [47]. Further investigations in the interplay of neurotransmitters and medication as well as enzyme activation patterns in certain areas of the brain are needed to better understand psychiatric disorders like ADHD better. Uncovering pathway interactions may lead to help increase dopamine release as well as its messaging at postsynaptic neuron.

MPH has been shown to interact with guanidine (*N*^G^) groups of certain Arg residues in the dopamine transporter (DAT) of rat striatum [48]. Methylation of those Arg residues of DAT by PRMT to form asymmetrically *N*^G^-dimethylated DAT, i.e., ADMA-DAT, could attenuate the interaction of MPH with DAT. Whether MPH inhibits the activity of PRMT towards DAT and other proteins including histones remains to be investigated. Another reason for diminished ADMA concentrations in ADHD patients could be an elevated hydrolysis of ADMA by DDAH. In vitro studies suggest that DDAH activity is increased by the inflammatory cytokine IL-1β [49]. However, a recent case-control comparison revealed no differences of IL-1β plasma concentrations in untreated ADHD patients compared to healthy controls aged six to 12 years [50]. That the urinary excretion of SDMA, ADMA, and DMA, the latter being a major DDAH-catalyzed metabolite of ADMA, did not differ between the −MPH and +MPH groups of our study suggests no alteration of the activity/expression of PRMT and DDAH by MPH. No statistically significant differences were found between the −MPH and +MPH groups with respect to the total Arg-dimethylation (i.e., ADMA+DMA+SDMA) and to the balance between asymmetric and symmetric Arg-dimethylation in proteins (i.e., ADMA+DMA)/SDMA). Treatment of ADHD children with MPH seems not to alter the PRMT-catalyzed Arg-dimethylation in proteins. Yet, being whole-body Arg-dimethylation measures [31], it does not exclude the possibility of MPH-mediated Arg-dimethylation of DAT in pediatric ADHD. 

### 4.4. Potential Effects of MPH and ADMA on Systemic Growth in ADHD

In addition to being an endogenous inhibitor of NOS activity, ADMA is supposed to exert NO-independent effects [44]. Thus, ADMA was found to be negatively correlated with growth in juveniles and seems to be an important factor in growth control [51,52]. In a study on ADHD children treated for at least two years with MPH, first a diminution in expected weight, but not in height, had been observed, yet after a few years the growth in weight and height was found to be greater than predicted [53]. Infusion of MPH in young psychiatric adult patients was found to acutely stimulate growth hormone release [54]. As ADMA plasma concentration, stature, weight, and BMI did not differ in the −MPH and +MPH groups, ADMA is unlikely to control for growth in pediatric ADHD. A large epidemiological study reported that MPH-untreated ADHD children (aged six to 13 years) in France were taller and heavier for young children, but shorter and lighter for older children, suggesting an age-dependent growth dysregulation [55]. The age range for the ADHD children of our study (six to 16 years) is within the age range reported in the above-mentioned epidemiological study. The head circumference was by 1.7% smaller in the +MPH group than in the −MPH group (*p* = 0.047, Table 1), suggesting that MPH-treatment of the ADHD children for about one year resulted in a retardation of the head growth. It has been reported that, when MPH-treatment is first started, any slowing of growth is compensated later on [53].

### 4.5. Blood Pressure in Pediatric ADHD and Potential Effects of MPH

In our study, we found that ADHD children chronically treated with MPH had closely comparable DBP values, but their SBP values were higher compared to the ADHD children not treated with MPH. It is worth mentioning that the SBP values of ADHD children acutely treated with MPH were also higher than those of ADHD children treated with placebo [56]. Therefore, the higher SBP levels in our study are likely to have been induced by MPH. Interestingly, the acute SBP increase in MPH-treated ADHD children was found to be associated with improvement in attention performance in these children [56]. On the contrary, ADHD patients with stimulant medication might have a higher frustration rate and suffer from increased stress exposure, which could favor blood pressure dysregulation [57]. It is surprising that we found increased SBP levels in the +MPH group, while the synthesis of ADMA, a cardiovascular risk factor in adults, was not elevated. This observation is contradictory to our initial hypothesis. We stated earlier that the elevated nitrate and nitrite concentrations, especially in the +MPH group, might reflect a higher NO synthesis in ADHD patients. This might point to a compensative reaction to protect against changes in the cardiovascular system caused by MPH. In the presence of reactive oxygen species (ROS), notably of superoxide, NO bioavailability would decrease by the reaction of superoxide and NO to build peroxynitrite [58]. Accordingly, elevation of NO production together with increased ROS might not only have positive effects, as peroxynitrite is an extremely potent oxidative species [59]. In fact, it was shown that MPH triggers ROS production in various animal studies [60,61]. By contrast, a clinical study points to protective effects of MPH against oxidative stress, at least after 12 weeks of MPH medication [62]. All NOS isoforms including eNOS can produce both NO and superoxide [63]. To the best of our knowledge, the effect of MPH or its major metabolite ritalinic acid on NOS-dependent formation of NO and superoxide from Arg has not been reported thus far. Future studies are needed to perform more comprehensive analyses of ADHD patients in relation to the Arg/NO pathway as well as in vitro studies on direct effects of MPH and ritalinic acid on NOS-dependent formation of NO and superoxide.

### 4.6. Study Limitations

A limitation of our study is the relatively small number of newly diagnosed and MPH-treated ADHD children. A longitudinal study with a larger group of newly diagnosed ADHD children and MPH-medicated ADHD children could help to elucidate the relevance of lower plasma ADMA and higher plasma nitrite concentrations in ADHD. Such studies should also address potential associations of circulating ADMA and nitrite with clinical and intellectual measures including IQ. Additionally, we cannot exclude that children with medication suffered from a severe form of ADHD, which could have caused selection bias. In an ongoing study, we are investigating the Arg/NO pathway in adult ADHD subjects. It is conceivable that parameters of the Arg/NO pathway, notably circulating ADMA and nitrite, could serve as biomarkers for the generally assumed cardiovascular risk in ADHD after long-term MPH therapy. In our study we did not measure additional biochemical parameters such as cGMP as a major of NO-dependent activation of guanylyl cyclase. Although several pathways may contribute to cGMP, its measurement in plasma and urine may provide valuable information about the biological activity of the Arg/NO pathway in ADHD. 

## 5. Conclusions

Our ADHD children aged six to 13 years had lower plasma ADMA and higher plasma nitrate and nitrite concentrations than age-matched children without ADHD. ADHD itself was associated with lower ADMA synthesis and higher NO synthesis. MPH did not change the plasma and urinary ADMA concentration, suggesting no effect of MPH on whole-body ADMA synthesis in pediatric ADHD. MPH treatment was associated with higher plasma nitrite concentrations in the ADHD children, suggesting an induction of NOS expression and/or activity in NOS-expressing cells. This might reflect a compensatory mechanism to antagonize SBP-enhancing effects of MPH. The Arg/NO pathway is altered in newly diagnosed ADHD children. MPH medication enhances NO synthesis apparently independent of ADMA, a potent endogenous inhibitor of nNOS. Further clinical studies are urgently needed to elucidate the underlying mechanism and the cardiovascular safety of MPH in ADHD. 

## Figures and Tables

**Table 1:** Anthropometric characteristics of the attention deficit hyperactivity disorder (ADHD) children without (−MPH) and with (+MPH) methylphenidate (MPH) treatment.

	−MPH	+MPH	*p*
Number (*n*)	23	19	-
Age (years)	9.12 ± 2.40	9.67 ± 1.83	0.43
Female [*n* (%)]	5 (21.7%)	4 (21.1%)	0.96
Age at diagnosis (years)	7.93 (7.41–9.20)	8.67 (7.12–11.2)	0.57
Stature (cm)	134 (130–141)	135 (128–151)	0.70
Head circumference (cm)	53.8 ± 1.54	52.9 ± 1.13	**0.047**
Weight (kg)	31.0 (27.0–43.8)	32.7 (25.7–46.7)	0.94
BMI (kg/m^2^)	16.6 (15.6–21.2)	17.8 (15.6–20.6)	0.73
DBP (percentile)	60.4 ± 23.3	62.3 ± 19.6	0.77
SBP (percentile)	59.5 ± 26.6	76.8 ± 19.0	**0.03**
MAP (mmHg)	78.0 ± 7.36	80.2 ± 6.69	0.33
Heart rate (beats/min)	79.0 ± 13.8	79.6 ± 13.4	0.90

Abbreviations. BMI, Body Mass Index; DBP, Diastolic Blood Pressure; SBP, Systolic Blood Pressure; MAP, Mean Arterial Pressure. Normally distributed data (mean ±standard deviation) were calculated with *t*-test, non-normally distributed data (mean (25th–75th interquartile range)) with Mann–Whitney test. Significant *p*-values are displayed in bold.

**Table 2:** Creatinine-corrected urinary concentrations of ADMA, symmetric dimethylarginine (SDMA), dimethylamine (DMA), arginine (Arg), nitrate, and nitrite of controls and ADHD children without (−MPH) and with (+MPH) methylphenidate (MPH) medication.

	Control	−MPH	+MPH	*p*
ADMA (µM/mM)	7.3 (5.3–8.9)	6.2 (5.6–6.9)	5.6 (5.4–6.9)	0.07
SDMA (µM/mM)	Not available	8.1 (6.5–9.6)	8.0 (7.3–8.9)	0.88
DMA (µM/mM)	Not available	45.7 (40.5–59.6)	43.3 (27.1–68.8)	0.61
ADMA+DMA+SDMA (µM/mM)	Not available	60.7 (54.8–76.7)	60.7 (41.5–81.8)	0.54
(ADMA+DMA)/SDMA	Not available	7.3 (5.7–8.2)	5.2 (4.1–9.9)	0.32
Arg (µM/mM)	Not available	3.3 (2.7–3.7)	2.3 (2.7–3.7)	0.12
Nitrate (µM/mM)	117 (86.3–154)	124 (91.6–150)	131 (87.9–162)	0.77
Nitrite (µM/mM)	0.23 (0.11–0.45)	0.19 (0.14–0.48)	0.24 (0.12–0.31)	0.97

Non-normally distributed data (median (25th–75th interquartile range)) were calculated with Mann–Whitney test or Kruskal–Wallis test.

**Table 3:** Molar ratios of nitrate, nitrite and creatinine in plasma (P) and urine (U), and fractional excretion (FE) in controls and ADHD children without (−MPH) and with (+MPH) methylphenidate (MPH) medication.

	Control	−MPH	+MPH	*p*
P_NOx_R	19.9 (11.9–40.4)	20.9 (16.0–24.8)	16.0 (13.0–19.8)	0.14
U_NOx_R	877 ± 357	669 ± 372	689 ± 368	0.35
U_NO3_/P_NO3_	27.9 (8.5–36.9)	21.8 (17.8–28.1)	20.0 (15.5–24.0)	0.25
U_NO2_/P_NO2_	1.3 (0.8–1.4)	0.6 (0.5–0.9)	0.5 (0.4–0.7)	**0.002**
U_NOx_R/P_NOx_R	24.5 (11.6–29.2)	30.6 (19.0–60.7)	40.6 (27.5–54.2)	0.05
FE_NO3_ (%)	Not available	15.8 ± 4.5	14.2 ± 3.5	0.22
FE_NO2_ (%)	Not available	0.6 (0.2–1.2)	0.4 (0.2–0.6)	0.26

Abbreviations. MPH, methylphenidate; P_NOx_R = P_NO3_/P_NO2_; U_NOx_R = U_NO3_/U_NO2_; FE = (U_NOx_ × P_Crea_)/(P_NOx_ × U_Crea_) × 100. FE_NO2_/FE_NO3_ was not calculated for controls because P_Crea_ was not determined. Normally distributed data (mean ±standard deviation) were calculated with *t*-test or one-way ANOVA; non-normally distributed data (median (25th–75th interquartile range)) were calculated with Mann–Whitney test or Kruskal–Wallis test. Significant *p*-values are displayed in bold.

## Supplementary Materials

The following are available online at https://www.mdpi.com/2077-0383/9/1/175/s1, Table S1: Plasma concentrations of ADMA, arginine (Arg), nitrate, and nitrite of controls (Co) and ADHD children without (−MPH) and with (+MPH) methylphenidate (MPH) medication, Table S2: Age-corrected ADMA and nitrite plasma concentration (mean ± SD) in ADHD children without (−MPH) and with (+MPH) methylphenidate (MPH) medication, Table S3: Age-corrected ADMA (A) and nitrite (B) plasma concentration (mean_adj_ ± SEM) in ADHD children without (−MPH) and with (+MPH) methylphenidate (MPH) medication according to the IQ range of 50–100 and >100.
