# Supplementary material for: Enhanced Nitric Oxide (NO) and Decreased ADMA Synthesis in Pediatric ADHD and Selective Potentiation of NO Synthesis by Methylphenidate
Source: J Clin Med. 2020 Jan 8;9(1):175. doi: 10.3390/jcm9010175 (PMC7019361; doi:10.3390/jcm9010175)
Supplement: Supplementary file 1 [file jcm-09-00175-s001.pdf]

Supplement to:

Enhanced nitric oxide (NO) and decreased ADMA synthesis in pediatric ADHD and selective potentiation of NO synthesis by methylphenidate

Kathrin Jansen[1*], Beatrice Hanusch[1*], Saskia Pross[1,2], Erik Hanff[3], Kathrin Drabert[3], Alexander Bollenbach[3], Irina Dugave[1,4], Christina Carmann[1], Rainer Georg Siefen[1], Barbara Emons[5,6], Georg Juckel[5,6], Tanja Legenbauer[7], Dimitrios Tsikas[3#], Thomas Lücke[1#]

Table S1 Plasma concentrations (median [25th – 75th interquartile range]) of ADMA, arginine (Arg), nitrate, and nitrite of controls (Co) and ADHD children without (-MPH) and with (+MPH) methylphenidate (MPH) medication

	Control	-MPH	+MPH	Statistics	*P*
ADMA (µM)	0.57 [0.51-0.66]	0.50 [0.46-0.54]	0.49 [0.46-0.54]	Co/-MPH: z = -18.9<br>Co/+MPH: z = -19.4<br>-MPH/+MPH: z = 0.60	**.008**<br>**.01**<br>.99
Arg (µM)	73.8 ± 17.3	68.1 ± 14.3	65.2 ± 14.1	F(2,78) = 2.16	.12
Nitrate (µM)	30.8 [27.0-37.6]	49.9 [44.8-53.5]	53.0 [44.1-55.4]	Co/-MPH: z = 23.1<br>Co/+MPH: z = 25.2<br>-MPH/+MPH: z = -2.17	**< .001**<br>**< .001**<br>.99
Nitrite (µM)	2.12 [0.83-2.32]	2.44 [2.13-2.8]	3.1 [2.78-3.5]	Co/-MPH: z = 16.7<br>Co/+MPH: z = 35.8<br>-MPH/+MPH: z = -19.1	**.01**<br>**< .001**<br>**< .001**

Normally distributed data (mean ± ~~SD~~standard deviation) were calculated with one-way ANOVA; non-normally distributed data (median [25th – 75th interquartile range]) with Kruskal-Wallis test; corresponding test statistics: *F*-value (between group df, within-group df), *z*-score. Arg, Arginine; ADMA, Asymmetric dimethylarginine.

Table S2 Age-corrected ADMA and nitrite plasma concentrations in ADHD children without (-MPH) and with (+MPH) methylphenidate (MPH) medication

	**-MPH**		**+MPH**			
	**Mean$_{adj}$ ± SEM**	**95% CI**	**Mean$_{adj}$ ± SEM**	**95% CI**	**Statistics**	***P***
ADMA (µM)	0.50 ± 0.01	[0.48; 0.53]	0.51 ± 0.01	[0.48; 0.54]	F(1,37) = 0.82	.77
Nitrite (µM)	2.67 ± 0.15	[2.38; 2.97]	3.16 ± 0.16	[2.84; 3.48]	F(1,39) = 5.15	**.03**

One-way ANCOVA adjusted for age. ADMA, Asymmetric dimethylarginine; Mean$_{adj}$, adjusted mean; SEM, standard error of the mean; *F*-value (between group df, within-group df).

Table S3 Age-corrected ADMA and nitrite plasma concentration (mean$_{adj}$ (SEM) in ADHD children without (-MPH) and with (+MPH) methylphenidate (MPH) medication according to the IQ range of 50 – 100 and above 100 (>100)

	**-MPH**		**+MPH**			
**IQ**	**50-100 (*n* = 3)**	**>100 (*n* = 8)**	**50-100 (*n* = 9)**	**>100 (*n* = 5)**	**Statistics**	***P***
ADMA (µM)	0.46 (0.02)	0.51 (0.01)	0.53 (0.01)	0.46 (0.02)	MPH: F(1,20) = 0.41<br>IQ: F(1,20) = 0.17<br>MPH×IQ: F(1,20) = 12.4	.53<br>.68<br>**.002**
Nitrite (µM)	2.45 (0.40)	3.01 (0.24)	3.10 (0.22)	3.22 (0.27)	MPH: F(1,21) = 2.14<br>IQ: F(1,21) = 1.12<br>MPH×IQ: F(1,21) = 0.53	.16<br>.30<br>.47

Two-way ANCOVA adjusted for age. ADMA, Asymmetric dimethylarginine; Mean$_{adj}$, adjusted mean; SEM, standard error of the mean; *F*-value (between group df, within-group df).
